# Annotations

**Published:** 1908-12-26

**Authors:** 


### Annotations.

### The Cambridge Water-supply.

Only too often have Local Government Board inquiries into the water supplies of towns or districts followed on disastrous epidemics of some water-borne disease. This time it is matter for congratulation that a water-supply containing the elements of calamity has been investigated in time. In the report of Dr. Thomson and Mr. Crosthwaite recently presented to the Board upon the water furnished by the Cambridge University and Town Waterworks Company, the conclusion is expressed that, while the greater part of the company's supply is obtained from unexceptionable sources, there is one portion of it, derived from the lower chalk, which is, under present conditions, dangerous to the lives and health of those who consume it. They discuss prevention of, and remedy for, this pollution; and after a highly technical and searching inquiry, conclude that both are impracticable. They recommend, therefore, that the whole of that part of the water-supply derived from the lower chalk be abandoned, and that its place be taken by an additional levy from the green-sand or from sources in the chalk not exposed to pollution. The investigators expressly declare that they find no evidence of any actual evil consequences having been so far experienced from the consumption of this water, but they very properly point out that there have been numerous instances of defective water-supplies producing no appreciable effect for years at a time. No doubt the authorities concerned will adopt the recommendations of the inspectors; though it is a little disquieting to see a letter from the Vice-Chancellor of the University in the papers, wherein he argues that because no harm has happened the water-supply must be satisfactory. We sympathise fully with his deprecation of panic, but not at all with his argument, which the inspectors had already been at particular pains to refute.

### Medical and Dental Protection.

On the unsatisfactory provisions of the Medical Acts in so far as they relate to the suppression of impostors and in so far as they do not relate to the prohibition of unqualified practice we have already commented (The Hospital, December 5, 1908, p. 235). The Dentists Act, though very much less perfect than it might be, at least avoids some of the more glaring defects of the Medical Acts, and we are glad to note that its provisions are now in a fair way to be carried out with greater success than hitherto. It has been established in the High Court that any statement implying training in dentistry or skill in extraction constitutes a description implying the possession of a legal qualification. This decision removes all possibilities of doubt such as have hitherto been felt and expressed by magistrates when dealing with offences under this Act. It appears that, on the strength of the decision in Barnes *v.* Brown, the London and Counties Medical Protection Society, which admits dentists as well as medical men to its ranks, intends shortly to institute proceedings against large numbers of quack dental practitioners. In congratulating dentists and the British Dental Association on the result of this litigation, we wish them all success in their further efforts to stop a very considerable evil. At the same Council meeting of the London and Counties Society at which these intentions were announced it was also pointed out to the medical profession that those members who attend workmen suffering from injuries within the scope of the Compensation Acts can recover fees from the respective employers or insurance companies only when the former have asked them to attend and have guaranteed the fees. Only too often in such cases have practitioners attended for prolonged periods in the belief that employers are liable, to find out months afterwards that they have no legal claim on anyone but the patients, who, having spent their compensation money, are not worth powder and shot.

### Old Fables and New Fancies.

Among existing facilities for relieving the itch for writing are several minor publishing agencies with rather high-flown names, a fair comment on whose productions is that in them great or technical subjects are dealt with by untrained capacities. Once in a while, perhaps, some originality of thought is discovered, but if so, at the cost of a heavy output of rubbish. Not unnaturally, the tone of the authors contributing to these presses, which are now noticeably growing in number, is decidedly revolutionary. No knowledgeable person would expect for a moment to find in their work respect for authority, or signs of strict editorship. Remembering all this, we are nevertheless astonished by a little book hailing from one of these associations which lately came under our notice. It treats of certain aspects of maternity in the magniloquent vein to be anticipated from its title: "The Bar of Isis: The Law of the Mother." Part I. informs the reader of another law, a fundamental cytological one (quite unknown, by the way, to timid dabblers like Boveri and Sutton), that fertilisation and fermentation are identical. And further on, it appears that the *vernix caseosa*, far from being a natural protective and lubricant, can give rise to serious infantile complaints; its cause being deducible from the fact that it is absent in the child of a woman who practises continence during pregnancy. The authority for this last statement, at all events, is not very recondite. Every medical student taking out his course of midwifery becomes familiar enough with these unedifying fables. But it is a little diverting to find them amongst the stock-in-trade of writers who are nothing if not pioneers; to see, in Swift's phrase, "histori-theophysiologically considered"—the narrations of Mrs. Gamp!

